# Middle Ear Disorders in Children with Down Syndrome: The Detrimental Effect on Speech and Language Development

**DOI:** 10.3390/children12050558

**Published:** 2025-04-25

**Authors:** Ariel Tenenbaum, Avraham Ben Yaakov, Yair Peled, Malena Cohen-Cymberknoh, Diana Averbuch, Ronit Brodie, Menachem Gross

**Affiliations:** 1 Department of Pediatrics, Hadassah-Hebrew University Medical Center, Jerusalem 91120, Israel; tene@hadassah.org.il (A.T.); yair@peledconsulting.co.il (Y.P.); malena@hadassah.org.il (M.C.-C.); adiana@hadassah.org.il (D.A.); 2 School of Medicine, Hebrew–University, Jerusalem 91120, Israel; 3 Department of Otolaryngology–Head & Neck Surgery, Hadassah-Hebrew University Medical Center, Jerusalem 91120, Israel; avrahambn@hadassah.org.il; 4 Department of Surgery, Hadassah-Hebrew University Medical Center, Jerusalem 91120, Israel; ronitu@hadassah.org.il

**Keywords:** down syndrome, otitis media, hearing loss, speech development, middle ear, developmental delay

## Abstract

**Objectives**: Our aim was to determine the prevalence of middle ear disorders and associated risk factors that contribute to speech and language developmental delays in children with Down Syndrome. **Methods**: A prospective, cross-sectional study was conducted in 70 children with Down Syndrome between the ages of 1 and 18. Data, including complete physical and otorhinolaryngological examinations, hearing tests, and evaluation of speech and language skills, was compared to a historic typical control group. **Results**: Recurrent otitis media was significantly higher in the study group (56.5%) compared to the control (26%) (*p* = 0.05). The rate of conductive hearing loss was significantly higher in the study group (71.2%) compared to the control (14.9%) (*p* = 0.0001). Conductive hearing loss was statistically associated with delayed speech development (*p* = 0.046). **Conclusions**: Middle ear disorders are common in children with Down Syndrome and are associated with conductive hearing loss and delay of speech and language development. This study emphasizes the importance of routine examination of this population by an Otorhinolaryngologist and speech therapist.

## 1. Introduction

Certain otorhinolaryngological (ORL) disorders are known to contribute to cognitive and developmental delay of speech. Among these are hearing impairments, frequently caused by recurrent ear infections or chronic middle ear effusion (CMEE). Children with Down Syndrome (DS) are at increased risk for many ORL illnesses, and as their intellectual capacity is compromised to begin with, the developmental consequences may be particularly grave. Thus, there is great significance for the early identification of ORL disorders in this population.

Children with DS have several predisposing factors that increase the incidence of infections. In addition to an immature immune system, they also have Eustachian tube dysfunction, a contracted nasopharynx, and narrow external auditory canals, conducive to frequent blockage by ear wax [1,2,3].

Ear infections are not uncommonly accompanied by conductive hearing loss (CHL) [4]. A survey of the frequency of hearing loss in typical children in the United States found it to be relatively high, with 14.9% diagnosed with a hearing impairment, although in most cases, only a minor deficit was detected [5]. In children with DS, the prevalence of hearing loss reaches as high as 38–78% and may be secondary to inadequate treatment of prolonged ear infections [1,6,7,8,9,10,11,12]. While hearing loss is a well-known risk factor for speech delay in typical children, the exact effect of hearing impairment on speech development in children with DS is not as clear. This population commonly has a baseline developmental delay, so the contribution of hearing loss is more difficult to determine. In addition to their distinct physical challenges, children with DS struggle with speech and language [13]. These obstacles are typically larger than expected for their degree of intelligence. Difficulties in speech and language have a significant and ultimate negative impact on the communicative skills of subjects with DS [14].

Unlike typical children, CMEE and hearing loss persist as children with DS age; therefore, continued surveillance of otologic and audiological status in these children is recommended. Early hearing loss can delay speech and language milestones in children with DS. Previous research indicates that children with hearing impairments exhibit delays in receptive vocabulary, expressive language, and speech accuracy compared to peers with normal hearing. For instance, children with hearing loss were, on average, seven months delayed in receptive vocabulary and comprehension and 24% less accurate in speech production [15]. Furthermore, a study assessing language outcomes in 41 children with DS found significant differences between those with early hearing loss and those with normal hearing. Children with hearing difficulties scored lower on measures of language expression and comprehension, receptive vocabulary, narrative tasks, and speech accuracy [16].

Assessment of children with DS is often complicated due to the wide spectrum of presenting deficits, making accurate assessment of their communication a difficult task. A balanced assessment consists of a variety of methods, including observations of the child as well as direct interactions in the form of standardized, informal, and dynamic assessments. Depending on the child’s age and level of abilities, a variety of assessment measures may be applicable to test DS child in the domains of content (vocabulary), form (grammar/syntax), and use (pragmatic language). For children over 3 years of age whose linguistic abilities are just emerging, a vocabulary inventory was conducted. This allows for comparing children with DS features in their development as compared to typically developing peers. For older, more verbal children who are using words, phrases, and/or sentences to express themselves, adapted standardized measures can be used.

The purpose of this study is to evaluate the prevalence of middle ear disorder in children with DS and to assess its effects on speech development. In addition, this study stresses the importance of ongoing ORL screening in children with DS.

## 2. Materials and Methods

Participants: Of 100 patients, the data from 70 children with DS who were in the care of the DS Center for Children at a tertiary medical center were retrospectively evaluated for this study over the course of 4 years. Exclusion criteria included patients with incomplete data (missing hearing screening/missing speech evaluations) and patients who withdrew from follow-up at the DS Center. In this multidisciplinary DS care center, children are evaluated by a pediatrician, an ORL specialist, and a speech therapist as part of the general follow-up.

Measures: A complete medical history was recorded for each child, including age, gender, history of gestation and delivery, growth and development, nutrition, and general medical issues. The number of siblings and family history were also recorded. Each child’s history of ORL disease was documented, including recurrent otitis media (at least three episodes of acute otitis media (AOM) in a period of 6 months, or four or more episodes in 12 months), CMEE (defined as the presence of middle ear effusion for more than three months’ time, occurring at any age on evaluation of the patient), hearing loss, recurrent pharyngotonsillitis, recurrent upper respiratory tract infections, indication for myringotomy or tympanostomy tubes, and any history of hospitalizations or surgeries, particularly adenotonsillectomy.

All children underwent complete physical examination by a pediatrician and an ORL specialist. The assessment was to determine the presence of tympanostomy tubes, signs of inflammation of ear canals and/or pharynx, size of the tongue, enlargement of tonsils, and nasal congestion.

All children were evaluated by two professional, experienced speech clinicians who belong to the DS Center for Children. The speech and language assessment was based on age-appropriate milestones. This evaluation consisted of documentation of specific parameters. History and acquisition of language developmental milestones, which belongs to the language developmental test that evaluates both receptive (understanding) and expressive (speaking) skills, was evaluated by the standardized preschool language scale 5 (PLS-5) (from birth to 7 years). Assessment of speech abilities, particularly the use of complete sentences (expressive language), was also assessed by the PLS-5 test. In addition, expression (expressive language) and comprehension (receptive language) were similarly measured by the PLS-5 test. Furthermore, subjects were evaluated for speech disorders, including stuttering, articulation disturbances, phonemic disorders, and lisps movements. Subjects were evaluated for eating or feeding disorders, including motor examination of the mouth, tongue, teeth, gums, and cheeks. Radiologic evaluation of the swallowing mechanism was completed in cases where necessary. Hearing evaluation included audiometry and tympanometry. For those very young children, hearing was evaluated via behavioral testing and testing via SDT/SRT (speech detection threshold/speech reception threshold) on bone.

### 2.1. Statistical Analysis

The data were analyzed using descriptive statistical methods. Differences between the study group and historic healthy control groups were analyzed using the χ^2^ test for categorical variables [4,5]. The association between categorical variables in the study group was analyzed using the χ^2^ test or Fisher’s exact test. The association between categorical variables and numeric variables was analyzed using the independent *t*-test. Statistical significance was accepted at a level of *p* ≤ 0.05. Statistical tests were performed using SPSS-25 software.

### 2.2. Ethical Approval

The study protocol was approved by the Hadassah Medical Center ethics committee (HMO-0507-09 and HMO-0665-17).

## 3. Results

Demographic characteristics of the study group are presented in Table 1. From an ethnic point of view, the Jewish population was predominant (86%). The mean age of the study group was 6.13 (age range 1–18 years). There were 36 (51.4%) boys and 34 (48.6%) girls. One family failed to cooperate with the interview.

The prevalence of recurrent otitis media and CHL in the study group as compared to the typical population is shown in Table 2. The rate of recurrent otitis media was significantly higher in the study group (56.5%) compared to typical children in the general population (26%) [4] (*p* = 0.05). The rate of CHL was significantly higher in the study group (71.2%) compared to typical children in the general population (14.9%) [5] (*p* = 0.0001).

The association between CHL and recurrent ear infections as well as language development, are demonstrated in Table 3. Recurrent otitis media was statistically associated with CHL (*p* = 0.001). However, no statistically significant association was found between recurrent ear infections and adenoidal enlargement, gender, age group, past hospitalizations, or number of siblings. Speech development was assessed through the use of complete sentences; those who did not speak in complete sentences after three years of age were defined as having a developmental delay. CHL was statistically associated with delayed speech development, as only 38.5% of subjects who had CHL spoke in complete sentences, as compared to 61.5% of subjects with normal hearing (*p* = 0.046).

## 4. Discussion

Children with DS are affected by multiple medical problems, several of which have a negative impact on their cognitive development. The added speech delay resulting from CMEE and CHL raises special concern in light of the pre-existing cognitive challenges inherent to DS.

This study demonstrated a high prevalence of recurrent otitis media in children with DS. It also has shown an increased prevalence of CHL in this population and a significant association with recurrent otitis media and CHL. These findings are all in line with previous studies [1,4]. In our study population, the majority of children with DS and CHL had delayed speech development. With other deficits faced by DS children, it is possible that chronic or recurrent hearing impairment compromises their speech and language learning environment and that a higher degree of hearing loss or prolonged periods of middle ear disorder is associated with poor outcomes on speech and language measures. These findings are consistent with other previous studies, such as those by Roberts et al., who found that language and communication development in children with DS, even with underlying mental delay, can be negatively affected by the presence of CMEE [17]. A retrospective study by Laws and Hall on 41 DS subjects aged 2 to 4 years, investigating the impact of early hearing loss on language outcome, showed significant differences between the group of DS subjects with hearing difficulties and DS subjects with more normal hearing. They concluded that early hearing loss has a significant impact on the speech and language development of children with DS [16]. As such, those authors also concluded that frequent hearing evaluation and screening by speech and language specialist is indeed necessary. Our study results further demonstrate the importance of early evaluation and treatment.

Hearing impairment, specifically conductive hearing loss, is likely to be an additional risk factor for further speech and language delay in DS pediatric subjects. In contrast to typically developing children, children with DS may not have the cognitive capacity to compensate for early hearing loss. Hearing impairment should be considered when managing speech and language deficits in children with DS.

With regard to intervention related to hearing status, the needs are clear: infants and children with DS will benefit from an aggressive treatment for hearing loss associated with middle ear disorder. Balkany, in 1980, identified three goals of such treatment: 1. Normalization of hearing through the insertion of tympanostomy tubes or hearing aids in some cases. 2. Interruption of the cycle of recurrent otitis media with effusion through the use of prophylactic medication. 3. Prevention of chronic ear disease through adequate otologic care [18].

Children with DS have a high prevalence of ORL problems, and when left untreated, these can lead to further morbidity, developmental delay, and impaired quality of life. A concerning trend was noticed in our study population; many of the problems were only first observed when the children arrived at our center—including older children. In our opinion, the various signs of ORL problems may erroneously be perceived by caretakers and under-informed medical professionals as inevitable and untreatable parts of DS when, in fact, ORL problems can be treated effectively.

### 4.1. Limitations of the Study

The study population consisted of children who were in the care of the Center for Children with DS. These children may not adequately represent the general population of children with DS, as they may come from families with relatively high compliance with medical follow-up and treatment. Alternatively, they may be children whose medical condition is relatively complicated to begin with, in which case they may actually experience more ORL disease than most children with DS. Additionally, the base range of IQ and extent of developmental delays of the children included in the study were not taken into consideration. As such, the significance of the effect of CMEE on such underlying delays was not able to be adequately determined.

### 4.2. Conclusions

Maintaining a high level of awareness of ORL illnesses in children with DS is imperative. Hearing screening tests should be performed routinely and in accordance with current recommendations [9,10,19]. If the child passes the newborn screening study, rescreen is recommended at 6 months of age for confirmation. In cases of external ear canal stenosis, an interval ear examination by an otolaryngologist every 3 to 6 months is recommended until the tympanic membrane is visualized and tympanometry is applicable. From 1 to 5 years, behavioral audiograms and tympanometry should be performed every 6 months until normal hearing levels are established bilaterally through ear-specific testing (behavioral audiogram or auditory brainstem response (ABR threshold)). External canal and middle ear diseases should be monitored by an otolaryngologist and treated promptly. Conductive hearing loss due to CMEE should treated by ventilation tympanostomy tubes. Conductive hearing loss that cannot treated surgically can be rehabilitated by hearing aids or soft-band with a bone anchoring hearing device (BAHD) until surgical management is suitable. From the age of 5 years, an annual ear-specific audiologic evaluation is recommended.

Effective, timely care for this population can be enhanced by providing follow-up and treatment under one roof by a staff trained to be fully attentive to these unique issues for this patient population. We believe that an extensive evaluation of possible ORL disorders is essential for all children and adults with DS. Furthermore, an experienced and dedicated ORL specialist should be a part of the multidisciplinary medical team that operates a medical center for the DS population. Further prospective studies are required to evaluate the correlation between hearing impairment and speech and language development in DS children.

## Figures and Tables

**Table 1:** Demographic characteristics of the study group.

Parameter		Patients N = 70 (%)
Gender	Male	36 (51.4)
	Female	34 (48.6)
Ethnicity	Jewish	60 (86)
	Arab	10 (14)
Age Group (Yrs)	1–2	17 (24.3)
	3–7	29 (41.4)
	8–18	24 (34.3)
Mean age (Yrs)	6.13	

**Table 2:** Prevalence of recurrent otitis media and conductive hearing loss in the study group compared to normal historic healthy control group [4,5].

Child’s Condition		Observed Prevalence in Study Group	Expected Prevalence [4,5]	*p* Value
Recurrent otitis media	No	30/69 (43.5%)	74%	*p* = 0.05
	Yes	39/69 (56.5%)	26%	
Conductive hearing loss	No	19/66 (28.8%)	85.1%	*p* = 0.0001
	Yes	47/66 (71.2%)	14.9%	

**Table 3:** Association between conductive hearing loss and recurrent otitis media and language skills as developmental delay.

	Hearing Loss N (%)		*p* Value
Recurrent otitis media	No	Yes	*p* = 0.001
No	14 (73.7%)	13 (27.7%)	
Yes	5 (26.3%)	34 (72.3%)	
Language skills	No	Yes	*p* = 0.046
Syllables to a few words	5 (26.3%)	14 (73.7%)	
Complete sentences	8 (61.5%)	5 (38.5%)	

## Data Availability

The original contributions presented in this study are included in the article. Further inquiries can be directed to the corresponding author.
